# Co-option of a coordinate system defined by the EGFr and Dpp pathways in the evolution of a morphological novelty

**DOI:** 10.1186/2041-9139-4-7

**Published:** 2013-03-01

**Authors:** Barbara MI Vreede, Jeremy A Lynch, Siegfried Roth, Élio Sucena

**Affiliations:** 1 Instituto Gulbenkian de Ciência, Rua da Quinta Grande 6, Oeiras, Portugal; 2 Departamento da Biologia Animal, Universidade de Lisboa, Faculdade de Ciências, Lisbon, Portugal; 3 Institute for Developmental Biology, Biocenter, University of Cologne, Zülpicher Strasse 47b, Cologne, Germany; 4 Present address: Dept. of Biological Sciences, University of Illinois at Chicago, 900 S. Ashland Avenue, Chicago, IL, USA

**Keywords:** Evolutionary novelty, *Drosophila melanogaster*, *Ceratitis capitata*, Oogenesis, Pattern formation, Dorsal appendages, Genetic network

## Abstract

**Background:**

Morphological innovation is an elusive and fascinating concept in evolutionary biology. A novel structure may open up an array of possibilities for adaptation, and thus is fundamental to the evolution of complex multicellular life. We use the respiratory appendages on the dorsal-anterior side of the *Drosophila* eggshell as a model system for morphological novelty. To study the co-option of genetic pathways in the evolution of this novelty we have compared oogenesis and eggshell patterning in *Drosophila melanogaster* with *Ceratitis capitata*, a dipteran whose eggs do not bear dorsal appendages.

**Results:**

During the final stages of oogenesis, the appendages are formed by specific groups of cells in the follicular epithelium of the egg chamber. These cells are defined via signaling activity of the Dpp and EGFr pathways, and we find that both pathways are active in *C. capitata* oogenesis. The transcription factor gene *mirror* is expressed downstream of EGFr activation in a dorsolateral domain in the *D. melanogaster* egg chamber, but could not be detected during *C. capitata* oogenesis. In *D. melanogaster*, *mirr*or regulates the expression of two important genes: *broad*, which defines the appendage primordia, and *pipe*, involved in embryonic dorsoventral polarity. In *C. capitata*, *broad* remains expressed ubiquitously throughout the follicular epithelium, and is not restricted to the appendage primordia. Interestingly *pipe* expression did not differ between the two species.

**Conclusions:**

Our analysis identifies both *broad* and *mirror* as important nodes that have been redeployed in the *Drosophila* egg chamber patterning network in the evolution of a morphologically novel feature. Further, our results show how pre-existing signals can provide an epithelium with a spatial coordinate system, which can be co-opted for novel patterns.

## Background

Classically, the concept of evolutionary novelty is that of a new trait, usually an anatomical or morphological one, that opens up the possibility of a wide adaptive radiation into new niches [1]. This definition places an emphasis on adaptation and is thus illustrative of the central role novel traits may have on shaping life on earth. Yet, it is a restrictive definition in that it implies knowledge of the adaptive value of the trait, eliminating traits that have been phylogenetically validated as novelties but lack ecological context. Moreover, this definition disregards the ontogenic aspects of the new trait, particularly of novel morphologies, the most prevalent type of novelty reported. An alternative definition has been proposed by Müller and Wagner [2] to a great extent circumventing the limitations described above. They define a morphological novelty as ‘a structure that is neither homologous to any structure in the ancestral species nor homonomous to any other structure of the same organism’ [2]. Still, this definition is not without problems, as it dislocates the problem of defining novelty to the definition of homology, which is another particularly elusive concept in biology [3-5]. Or, as phrased by Moczek [6]: ‘our definition of novelty now only becomes as strong as our definition of homology’. Nonetheless, and as new perspectives and conceptual contributions to this debate arise [7], in the confined context of this paper we will adopt this latter, more operational definition of a morphological novelty.

At the mechanistic level, one of the most important contributions of evo-devo to our understanding of the evolutionary process has been the refinement and experimental validation of the gene recruitment concept (co-option). These are key innovations at the genetic level that may underlie differences in cellular growth and morphogenetic processes between related organisms, which have diverged morphologically [8]. In recent years many examples have demonstrated that evolution largely relies on recycling old genes and pathways to generate novel patterns and morphologies [9,10].

**Figure 1:**
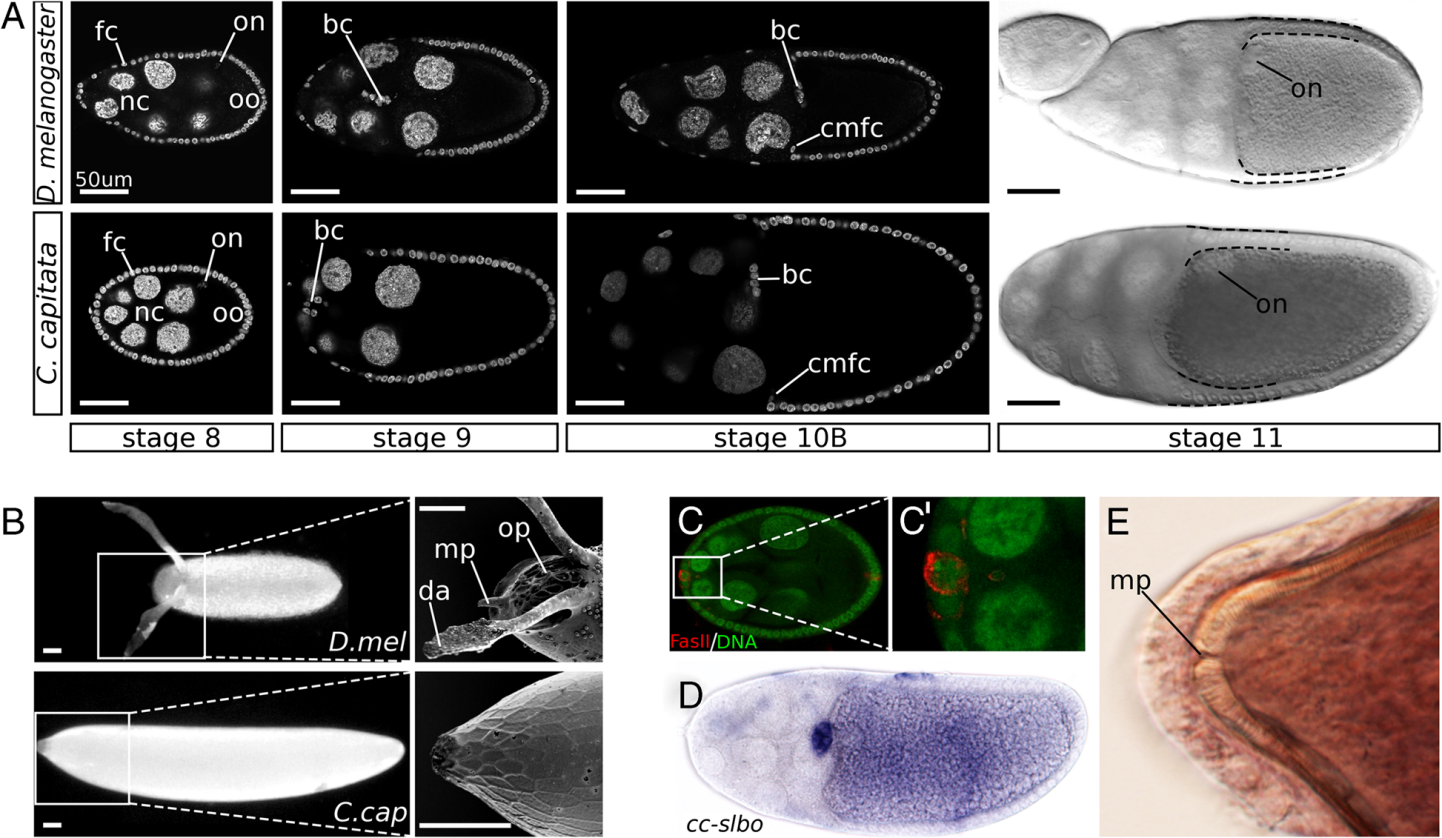
***C. capitata*is a useful species for comparison with *D. melanogaster.*** Posterior is to the right; scale bar is 50 μm. (A) Stages of oogenesis were identifiable in C. capitata using criteria described in D. melanogaster. At stage 8 of oogenesis, the oocyte nucleus (on) is localized asymmetrically in the oocyte (oo), which at this stage is of roughly equal size to the nurse cells (nc). At stage 9 the follicle cells (fc) start their migration to posterior: anterior follicle cells stretching over the nurse cells, and posterior follicle cells forming a layer of columnar cells over the oocyte. At the same time, a cluster of border cells (bc) migrates between the nurse cells to the anterior end of the oocyte. Late stage 10 sees the columnar follicle cells migrating centripetally (cmfc), in between nurse cells and oocyte. Stage 11 shows a difference between D. melanogaster and C. capitata egg chambers in the relative thickness of dorsal and ventral follicle cell layers. (B) Eggs of D. melanogaster and C. capitata, the former bearing obvious structures: dorsal appendages (da), operculum (op), and an outward micropyle (mp). (C) Fas-II staining of stage 8 C. capitata egg chamber, identifying the polar cells, part of the border cell cluster. (D) *In situ* hybridization with a probe against slbo confirms the identity of the border cell cluster in the C. capitata egg chamber. (E) A small pore is visible in the newly formed eggshell of C. capitata, likely a structure homologous to the micropyle (mp).

The model *Drosophila melanogaster* has often been criticized for being extremely derived, and therefore a poor reference in understanding the prototypical insect. Here, we turn this argument around and use *D. melanogaster* as a source of novelty by identifying a novel morphological feature acquired in the evolution of the Drosophilidae family: the egg dorsal appendages (Figure 1B). The formation of these dorsal-anterior chorionic filaments during *Drosophila* oogenesis has already been used as a model system for the study of many developmental mechanisms, such as epithelial patterning [11,12], and tube formation [13-15].

Most (though not all) eggs of Drosophilidae bear dorsal appendages, which are thought to have a single origin in their last common ancestor [16]. The appendages are hollow tubes protruding from the dorsal-anterior end of the chorion, and provide an oxygen supply to the immersed egg [16,17]. They portray a striking diversity within the Drosophilidae family [18-20], which makes the appendages an interesting subject from an evolutionary perspective. The adaptive advantage of respiratory appendages is emphasized by Hinton [16]: they allow the egg to increase its oxygen-absorbing surface without risking desiccation. Indeed, similar eggshell structures have evolved independently at least 11 more times within Diptera, and at seven more instances in other insects [16,17]. Nonetheless, and despite their assumed evolutionary advantage, they are not so prevalent that a single origin of these structures in all Diptera seems likely.

In addition to the dorsal appendages, the Drosophila egg carries an operculum and a micropyle: structures relevant for hatching and fertilization, respectively (Figure 1B). These structures are formed during the last stage of oogenesis by designated cells in the follicular epithelium that change shape prior to the deposition of chorionic proteins [13,21]. Specification of the appendage primordia occurs chiefly through the activity of two main signaling pathways: EGFr and Dpp [13,22].

### EGFr and Dpp signaling define appendage primordia

**Figure 2:**
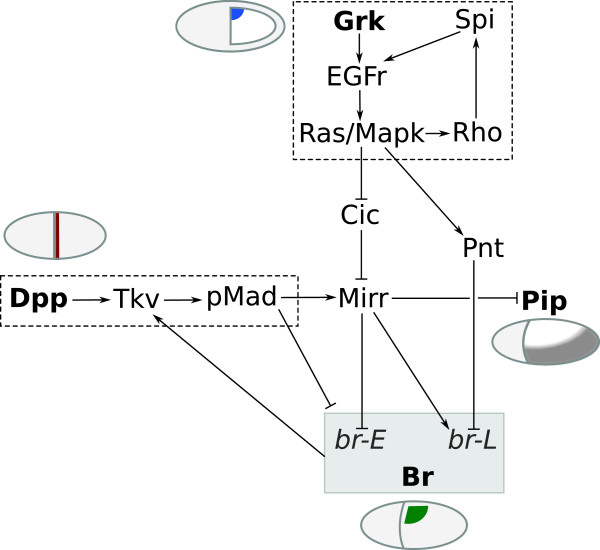
**A simplified representation of the genetic network underlying dorsoventral polarity (*pip*) and DA-formation (br) during *D. melanogaster *oogenesis.** Input comes from two main signaling pathways, EGFr and Dpp, providing dorsoventral and anteroposterior information, respectively, and results in the specification of domains on the epithelium expressing *pip* and br.

**Figure 3:**
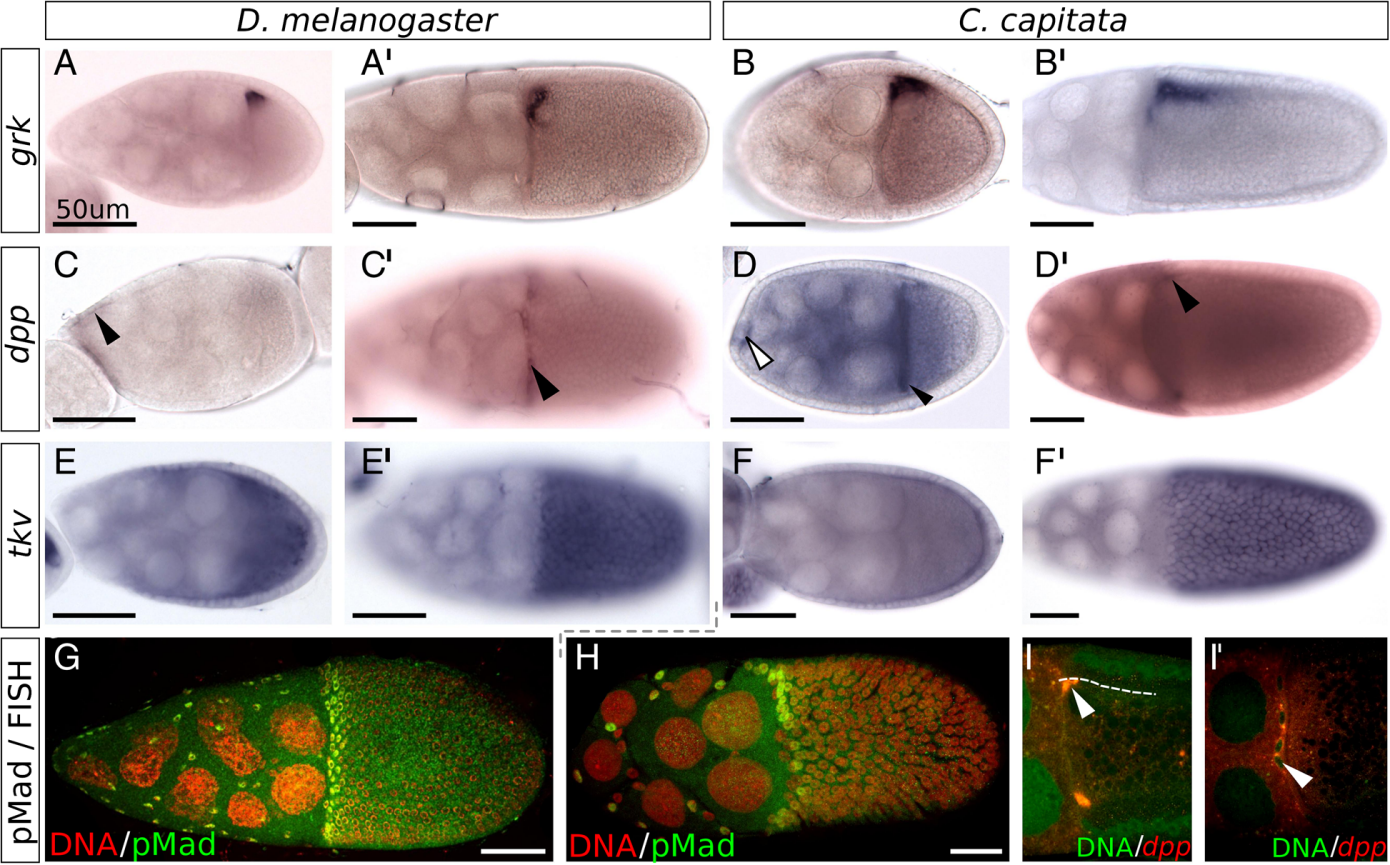
**Dpp and EGFr activity in *C. capitata *and *D. melanogaster *oogenesis.** Posterior is to the right, ventral to the bottom. (A to B’) The grk transcript localizes in the dorsal-anterior corner of the oocyte, both in D. melanogaster(A,A’) and C. capitata(B,B’). A and B are stage 8; A’ and B’ are stage 10B. (C to D’) Expression of dpp differs between the two species: (C)D. melanogaster dpp expression starts in a subset of anterior follicle cells at stage 8 (arrowhead). (C’) At stage 10A dpp is only seen in a ring of follicle cells at the border between the nurse cells and oocyte (arrowhead). (D) In a stage 8 C. capitata egg chamber, the dpp transcript is seen in the border cell cluster (empty arrowhead), the nurse cells, and localized anteriorly in the oocyte (black arrowhead). (D’) At stage 10, the transcript localizes in a ring at the anterior-outer edge of the oocyte (arrowhead), see also (I). (E to F’) Expression of thickveins in all follicle cells of stage 9 (E,F) and early stage 10 (E’,F’) egg chambers of both species. (G to H) In both D. melanogaster(G) and C. capitata(H) stage 10A egg chambers, activation of the Dpp pathway, visualized with immunohistochemistry against pMad, occurs in the stretched follicle cells overlying the nurse cells, and a few anterior rows of columnar follicle cells. (I,I’) FISH of dpp in C. capitata. (I) In a stage 10A egg chamber the dpp transcript localizes just underneath the follicle cells (arrowhead; dashed line indicates border of follicle cells). (I’) In stage 11 expression can be seen in migrated follicle cells between the nurse cells and oocyte (arrowhead).

Pattern formation on the follicular epithelium occurs through the activation of a genetic network by two main input pathways: EGFr and Dpp signaling (Figure 2). Around stage 8 of Drosophila oogenesis, dorsal patterning is initiated when the TGF-α-like ligand Gurken (Grk) localizes to the dorsal-anterior corner of the oocyte (Figure 3A). Grk associates with the oocyte nucleus, which is pushed by microtubules to a dorsal-anterior position [23], breaking dorsoventral symmetry in the egg chamber [24]. The Grk signal then activates EGFr in the adjacent follicle cells, leading (directly and indirectly) to the expression of several transcriptional targets, among which are *mirr*or (*mirr*) [25,26], rhomboid (rho) [27], and pointed (pnt) [28] (Figure 2).

**Figure 4:**
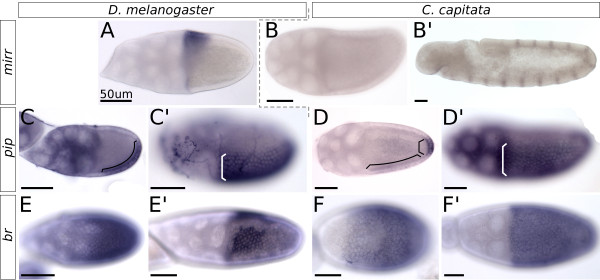
**Expression of *mirr*, *pip *and *br *in *C. capitata *and *D. melanogaster *oogenesis.** All images are *in situ* hybridizations; posterior is to the right, and ventral to the bottom. The scale bar is always 50 μm. (A)*mirr* expression in a stage 10 egg chamber of D. melanogaster. (B)*mirr* expression in a stage 10 egg chamber of C. capitata, with (B’) a positive control for the probe in the embryo. (C) Expression of *pip* in a stage 9 egg chamber of D. melanogaster shows dorsal and anterior repression of the gene, and an equal expression strength in ventral and posterior follicle cells (marked by bracket). (C’) Stage 10B shows the final stabilized *pip* pattern. (D) In C. capitata, ventral *pip* expression starts only at stage 10A, and is visibly lighter than the posterior domain (domains marked by separate brackets). (D’) At stage 10B the pattern has stabilized and shows the same sharp on-off boundary between cells expressing and not-expressing *pip* as seen in D. melanogaster(C’). (E) Expression of br is visible in all follicle cells of the D. melanogaster stage 9 egg chamber. (E’) Stage 10B shows br expressed in the roof cells of the appendage primordia. (F) In C. capitata, stage 9 expression is similar with all cells expressing br. (F’) A C. capitata stage 10B egg chamber shows how all follicle cells continue expressing br, and a pattern such as in D. melanogaster(E’) is not formed.

Meanwhile, Dpp signaling starts at stage 8 with the expression of dpp in a subset of anterior follicle cells [29] (Figure 3C). Dpp protein diffuses to more posterior follicle cells, forming a morphogen gradient. It acts via the receptor Thickveins (Tkv) in the follicular epithelium to phosphorylate Mothers Against Dpp (Mad), activating the pathway in a graded manner [30]. Dpp has also been suggested to be required for the expression of *mirr*[31], which starts at stage 10A in a wide dorsoanterior domain (Figure 4A). Recent work by Fuchs et al. [32] shows how the transcription factor *mirr*, regulated by both Dpp and EGFr activity, and the ETS-domain transcription factor Pnt, expressed in a more narrow stripe along the midline, subsequently establish two groups of cells expressing broad (br) through two rounds of signaling. First, *mirr* represses br, which has been expressed in all follicle cells up to this point, in a wide dorsoanterior region through the brE enhancer. Then, br expression is upregulated again by *mirr*, but repressed by Pnt, through the brL enhancer (Figure 2). The two resulting patches of Br-positive cells on either side of the midline are identified as ‘roof cells’: they will later constrict apically and shape the roof of the appendage tube [33]. Adjacent to the Br-positive patches is a single L-shaped row of cells, bordering the anterior and the central edge of the roof domain. These cells express high levels of rho, and elongate directionally to form the floor of the tube [33]. rho expression is regulated mainly by activation of the EGFr pathway, which is highly dynamic throughout oogenesis, and shows the same L-shaped pattern at the definition of the floor cells [34]. Rho itself is involved in the dynamic EGFr activation as it cleaves the EGFr ligand Spitz (Spi) into its active form, thereby providing a positive feedback loop for EGFr signaling [27,35,36] (Figure 2).

Importantly, EGFr signaling also determines the dorsoventral axis of the future embryo [37]. Via *mirr*, *pipe* (*pip*) expression is restricted to the ventral follicle cells (Figure 2), leaving an asymmetric distribution of *pip* protein at the end of oogenesis [32,38-40]. *pip* is upstream of a proteolytic cascade in the embryo, leading to the well-known gradient of nuclear Dorsal that regulates the germ layers of the early embryo [41].

Dpp, too, is required for processes other than the specification of the appendage primordia. As the inward movement of the centripetally migrating follicle cells starts (Figure 1A), dpp is expressed in the leading edge of these cells, and disrupted Dpp signaling has been associated with defects in this migration [29]. Dpp is required furthermore for the formation of the operculum [29,42].

In summary, EGFr and Dpp activity specify dorsoventral and anteroposterior polarity in the epithelium, respectively, and their signaling information is integrated by Br and Rho, which together specify the appendage primordia. In addition, both signaling pathways are crucial for proper egg formation and further embryonic development, linking the formation of secondary (novel) structures to essential (thus presumably ancestral) developmental events.

### Ceratitis capitata

Considering the relatively novel acquisition of eggshell appendages in the family Drosophilidae, it is interesting to examine the underlying patterning network in the context of a fly species that does not possess these specialized structures. Tephritidae are estimated to be separated by about 65 million years of evolution from Drosophilidae [43]. For our comparison we chose a Tephritid fly that has been established as a laboratory organism: the Mediterranean fruit fly Ceratitis capitata. C. capitata is an agricultural pest, which has motivated widespread international research, including a genome project and the development of genetic tools [44-46].

In this study, we have examined both EGFr and Dpp signaling as well as their downstream targets in C. capitata oogenesis, in order to understand the genetic network patterning the follicular epithelium prior to the evolution of dorsal appendages. Determining which genes behave differently in the formation of appendage-bearing (D. melanogaster) and appendage-less (C. capitata) eggshells can help us understand the co-option of genes and the genetic network in the evolution of this novel feature. Our analysis points to a key role for the transcription factor *mirr*, both in its regulation and in its transcriptional targets. Furthermore, the activity of both the EGFr and the Dpp pathway in C. capitata oogenesis leads us to hypothesize that these pathways provided positional information to the ancestral follicular epithelium, which could have facilitated further downstream patterning required for the development of the dorsal appendages.

## Material and methods

### Fly maintenance

Our initial Ceratitis capitata culture was kindly (and repeatedly) provided by Andrew Jessup (IAEA Seibersdorf, Austria), originating from flies captured in Argentina. Adult flies were maintained on a diet of sugar and hydrolyzed yeast protein, and larvae were reared on a mixture of bran, sugar and yeast. All stages were maintained at room temperature. Drosophila melanogaster Oregon R. was maintained on regular fly food at room temperature.

### Cloning

Gene-specific sequences were isolated from C. capitata cDNA by PCR using degenerate primers (for dpp, *mirr*, rho and tkv), as well as C. capitata specific primers (for Cc-br, Cc-cic (capicua), Cc-grk, Cc-pnt, Cc-*pip* and Cc-slbo). Specific primers were designed using contigs from the C. capitata genome project, provided by the Medfly Whole Genome Sequencing Consortium (led by Drs Alfred Handler and Marc Schetelig, USDA, Agricultural Research Service, Gainesville, Florida; Giuliano Gasperi and Ludvik Gomulski, Department of Biology & Biotechnology, University of Pavia, Italy; and Stephen Richards and Steven Scherer, Baylor College of Medicine Human Genome Sequencing Center).

For Cc-*pip* two primer combinations were used, generating two separate probes for *in situ* hybridization. These probes were (1) against the common part of all *pip* isoforms, and (2) against Cc-*pip*-ST2, the homologue of Dm-*pip*-ST2 (isoform A). Corresponding probes were made for the positive controls in D. melanogaster.

The (partial) nucleotide sequences of all C. capitata genes used in this study have been deposited with GenBank, and are available under the following accession numbers: KC150010 (Cc-br), KC150011 (Cc-cic), KC150006 (Cc-dpp), KC150012 (Cc-grk), KC150007 (Cc-*mirr*), KC150013 (Cc-*pip*, common part), KC150014 (Cc-*pip*, specific to isoform ST2), KC150015 (Cc-pnt), KC150008 (Cc-rho), KC150016 (Cc-slbo) and KC150009 (Cc-tkv).

### Immunohistochemistry

Ovaries were dissected in cold PBS and fixed for 20 minutes at room temperature in 4% formaldehyde in PBTx (0.1% Triton-x100 in PBS). After fixation they were washed several times in PBTx-B (1% BSA in PBTx) at room temperature for 1 hour. Antibody incubation was done overnight at 4°C. The rabbit anti-pMad antibody was kindly provided by the laboratory of Ginés Morata (Centro de Biología Molecular Severo Ochoa, Autonomous University of Madrid, Spain), and was used at a concentration of 1 : 100 in PBTx-B. Anti-Fasciclin II (1D4) was obtained from the Developmental Studies Hybridoma Bank (maintained at the University of Iowa, Department of Biology, Iowa), and was used at a concentration of 1 : 50. Secondary antibodies (Alexa fluor 488/546 goat-anti-rabbit or anti-mouse IgG (H+L), Molecular Probes) were used at a concentration of 1 : 2000, overnight at 4°C. Nuclear staining was done with Dapi.

### *In situ* hybridization (ISH)

Ovaries were dissected in cold PBT (0.1% Tween-20 in PBS) and transferred to 4% paraformaldehyde in PBS, where they were fixed overnight. They were subsequently washed in PBS, dehydrated and stored in 100% MeOH at −20^∘^C. The protocol for ISH was taken from Tautz and Pfeifle [47] and modified for oogenesis. The main change concerned the adjustment of the proteinase K digestion to 10 minutes 50 μg/mL at room temperature.

To ensure identical conditions during the experiment, the positive controls with embryos were done in the same well as the ovaries, starting at the pre-hybridization incubation in hybridization buffer at hybridization temperature. This was done because the proteinase K treatment for ovaries is much harsher than the one we used for embryos (10 minutes 50 μg/mL vs. no proteinase K at all).

## Results

### C. capitata oogenesis is a suitable system for comparison

The C. capitata eggshell carries no structures that can be identified as homologues of the operculum, outward micropyle and dorsal appendages (Figure 1B). Still, it is not entirely symmetrical, both over the anteroposterior axis and the dorsoventral axis. The anterior end of the chorion shows markedly stronger imprints of (previously present) follicle cells when compared to the posterior end. While we cannot say with certainty which side is dorsal and which is ventral, it is clear that one is more convex than the other. As both late stage egg chambers (Figure 1A,D) and early embryos (data not shown) are clearly more convex at the ventral side, it is a reasonable assumption that the convex side of the egg is ventral.

From an initial observation of C. capitata oogenesis we can conclude first and foremost that it is a suitable system for comparison with D. melanogaster. C. capitata ovaries, like those of Drosophilidae, are meroistic polytrophic ovaries. While the egg chamber of C. capitata is usually larger than the corresponding stage in D. melanogaster, there is no notable difference in the number of cells that make up the follicular epithelium. Instead, the size of C. capitata follicle cells is increased with respect to those of D. melanogaster, thus contributing to a larger egg chamber as a whole (Figure 1A).

The structure of the egg chambers as well as the progression of stages is nearly identical to that of Drosophila, providing a good basis for comparison (Figure 1A). Starting at mid-oogenesis, we can observe the asymmetric localization of the oocyte nucleus (stage 8), as well as follicle cell migration (stage 9), and centripetal migration (stage 10B). Also visible is the dumping of nurse-cell content into the oocyte, as evidenced by the increasing size of the oocyte relative to the nurse cells, which disappear eventually. All these are important and stage-defining steps in Drosophila oogenesis. We will therefore refer to the stages defined in D. melanogaster [48] when describing C. capitata oogenesis.

In addition to the migration of the main body follicle cells, a cluster of anterior follicle cells can be seen to migrate between the nurse cells at stage 9. Their migration ends at the posterior edge of the nurse cells, adjacent to the oocyte, where they are shortly joined by the centripetally migrating follicle cells. In D. melanogaster these cells are known as border cells, and can be identified by the expression of slbo [49], as well as with the polar-cell-specific label Fasciclin II. Both markers confirmed the identity of the border cell cluster in C. capitata (Figure 1C,D). Interestingly, as the border cells have been associated in D. melanogaster with the formation of the micropyle, no obvious external micropyle can be seen on the C. capitata egg (Figure 1B). However, upon closer examination of the newly formed eggshell we found a pore-like structure on the anterior side of the eggshell, likely homologous to the micropyle pore (Figure 1E). This is consistent with the observed border cell localization in C. capitata, as these cells are known to form the pore of the micropyle, but not the outwardly visible structure [48].

### Both EGFr and Dpp pathways are active in C. capitata oogenesis

In C. capitata ovaries, the initial activation of the dorsoventral patterning cascade by the ligand Gurken occurs similarly to D. melanogaster. In the early stages, the Cc-grk transcript is visible in the oocyte at the anterior cortex (data not shown), and around stage 8 the pattern becomes restricted to the putative dorsoanterior side of the oocyte (Figure 3A,B). The transcript disappears around stage 11.

While we were unable to obtain patterns of EGFr activation because of practical difficulties, the fact that TGFα-EGFr signaling is conserved in insects as distant as Tribolium and Gryllus [50], functioning upstream of embryonic dorsoventral patterning even in drastically different systems of oogenesis, makes it unlikely that this would be any different in C. capitata. Indeed, we observed the dorsal repression of a known target of EGFr signaling in D. melanogaster: the gene *pip* (Figure 4D,D’).

In contrast with oogenesis in D. melanogaster, Cc-dpp is not expressed in the somatic follicle cells, but instead in the germ line. Expression of Cc-dpp is first visible as early as the germarium. Once the egg chamber is formed, the dpp transcript localizes to the oocyte. When the oocyte increases in size, the mRNA seems to accumulate at the putative anterior end of the oocyte, in a ring around the edge, adjacent to the follicle cells (Figure 3D,D’,I). Interestingly, this ring is reminiscent of the D. melanogaster pattern, where dpp is expressed in the stretched follicle cells as well as a few anterior rows of columnar follicle cells, resulting in a similar ring of dpp expression around the anterior end of the oocyte (Figure 3C’). The main difference, of course, is that the transcript is located in different cell types.

One exception to the exclusive germ line expression of Cc-dpp is the border cell cluster. This migrating group of anterior follicle cells is not known to express dpp in D. melanogaster, but is the only group of somatic cells during oogenesis to express Cc-dpp. Expression is visible around stage 8, when the cell cluster is defined (Figure 3D, empty arrowhead), and persists through migration until the edge of the nurse cells is reached.

A possible second group of Cc-dpp expressing follicle cells was identified using fluorescent *in situ* hybridization (FISH). This group of cells is centrally located between the nurse cells and the oocyte in late stage 11 (Figure 3I’). Due to the very small sample size we cannot say with certainty whether these cells are the border cells or part of the follicle cells that have centripetally migrated inwards. As the signal of Cc-dpp expression does not persist in the border cell cluster after migration is completed, the observation could either indicate a new round of Cc-dpp expression in this cluster should these cells indeed be border cells, or it could point to conservation of dpp expression in the leading edge of centripetally migrating follicle cells.

While expression of the ligand may differ somewhat between the two species, downstream signaling is remarkably similar. The expression of the homologue of the Dpp pathway type I receptor tkv is not visibly different in C. capitata from D. melanogaster: Cc-tkv is expressed in the follicular epithelium (Figure 3E,F), and disappears around stage 11 or 12. More importantly, the activity of the pathway, shown through immunohistochemistry for the phosphorylated form of Mad (pMad), is initially not different between the two species, despite the altered localization of the dpp transcript (Figure 3G,H).

Differences in Dpp pathway activation between C. capitata and D. melanogaster start around stage 10B, when expression of Dm-tkv becomes restricted to the Br-positive cells of the appendage primordia, naturally affecting pMad patterns [51,52]. These dynamics were not observed in C. capitata, where no Br-positive domains are formed (Figure 4F’).

### Patterning of the follicular epithelium downstream of EGFr and Dpp

The dynamics of EGFr and Dpp signaling and subsequent epithelial patterning in D. melanogaster egg chambers are key in defining the appendage primordia. Identifying the point in the genetic network where C. capitata no longer resembles D. melanogaster is therefore an important step in understanding the evolution of the dorsal appendages, as it could indicate the point where the network was co-opted.

Our first candidate for co-option was found when we saw that no expression of *mirr* could be detected in C. capitata egg chambers (Figure 4B). The probe against Cc-*mirr* did reveal clear expression in the C. capitata embryo, in a pattern familiar from expression in D. melanogaster (Figure 4B’) [53].

*mirr* regulates the transcription of br in those cells that will give rise to the dorsal appendages (Figure 4E’). Unsurprisingly, the br-positive domains do not appear on the C. capitata stage 10B follicular epithelium (Figure 4F’), nor during any other stage of oogenesis. Early expression of br could be seen uniformly in the follicular epithelium, as in D. melanogaster, but the late expression dynamics, both the dorsal-anterior repression and the appearance of the two domains, were not observed; instead, expression diminished around stage 11 and had disappeared entirely by stage 12.

Preliminary results indicate that two other genes relevant for D. melanogaster epithelial patterning do not play a role in the C. capitata dorsal-anterior epithelium: expression of pnt, encoding the transcription factor responsible for the midline repression of br, could not be detected in the dorsal-anterior follicular epithelium of C. capitata. A second known expression domain of pnt at the posterior pole of the egg chamber was clearly visible from an early stage (stage 8), providing a positive control for the *in situ* hybridization and the pnt probe (Additional file 1). Transcription of the gene rho was also not detected in either the early broad dorsoanterior domain, or in the late hinge-shaped patterns adjacent to the br expressing domains [22] (Additional file 1). However, as both early rho expression and the dorsoanterior domain of pnt can be difficult to detect in D. melanogaster egg chambers as well, we cannot be completely certain of the absence of pnt and rho transcripts in the dorsoanterior follicular epithelium of C. capitata.

### Conserved expression of *pip*

Interestingly, especially in the light of the absence of detectable Cc-*mirr* expression, Cc-*pip* is repressed dorsally: the transcript is expressed asymmetrically, and clearly localizes to the ventral follicular epithelium. In a similar dynamic—though not precisely identical—to D. melanogaster, Cc-*pip* expression starts at stage 8 in follicle cells at the posterior pole of the egg chamber (Figure 4D). This posterior expression domain during stages 8 and 9 is well known in D. melanogaster [38,40]. During early stage 10, ventral follicle cells start expressing Cc-*pip*, and by late stage 10 expression in ventral and posterior follicle cells is of equal strength (Figure 4D’). The pattern at this stage is identical to the expression pattern of Dm-*pip* (Figure 4C’), including the sharp on-off boundary between ventral and dorsal cells. These results were obtained using two separate probes: one against the common part of all *pip* isoforms, and one specific to the homologue of isoform A (or *pipe*-ST2), confirming that the same isoform is used in C. capitata oogenesis as is known to function in D. melanogaster [54].

## Discussion

### Pre-existing functional signals provide positional information

A first conclusion we can draw from the work presented is the fact that the activity during oogenesis of the two main patterning pathways, EGFr and Dpp, preceded the evolution of dorsal appendages and their underlying epithelial patterns [29]. The ancestral role of EGFr signaling lies in determining the dorsoventral axis of the future embryo [50], while Dpp is involved in various cell migrations required for the developmental progression of the egg chamber. Activity from these pathways provides the epithelium with positional information that may constitute an important facilitator for novel patterns to evolve.

A formalism for pattern formation on the Drosophila melanogaster follicular epithelium was developed in 2008 by Yakoby et al. [11]. They propose a combinatorial code of principle patterns from which all expression patterns at the dorsal-anterior follicular epithelium can be derived. As their formalism includes both the EGFr and Dpp input, as well as three additional primary building blocks specific to the dorsal-anterior epithelium, this system constitutes the next step of pattern formation in the evolution of Drosophila eggshell morphology. Interestingly, this specification of patterns from a system of higher order components is an emerging theme in regulatory evolution [55]. Our results therefore fit within the larger research theme of how pre-existing information may bias the future evolution of pattern formation and morphology.

### Upstream differences in Dpp signaling between D. melanogaster and C. capitata

Despite the fact that the pattern of Dpp activity is similar between C. capitata and D. melanogaster, the differences in the underlying expression of its ligand dpp are puzzling. Not only are there differences in the expression patterns of Dm-dpp and Cc-dpp, but the transcripts are produced by an altogether different cell type. Cc-dpp is likely expressed by the nurse cells and transported to the oocyte, both of which are germ line, while D. melanogaster requires dpp expression in the somatic follicle cells.

Several functions have been described for Dpp signaling in D. melanogaster [56]. In the context of oogenesis, the need for Dpp signaling in the formation of anterior eggshell structures has been clearly established: dpp is expressed in the cells that will form the operculum, and disruptions of Dpp signaling cause misplaced and deformed appendages [29,57-59]. Additionally, Dpp signaling is needed for the centripetal migration of follicle cells, to maintain structural integrity of the egg chamber, and for dumping of nurse cell content into the oocyte [29]. However, expression of the signaling molecule Dpp is only required in the somatic follicular epithelium: in D. melanogaster germ line dpp is not required during oogenesis [60]. In C. capitata, dpp is clearly expressed in the germ line, and the signal acts through receptors in the soma. While it cannot be ruled out that the Dpp activity in the follicular epithelium is a response to early dpp expression in the border cell cluster, the transcript in the nurse cells as well as the ring of dpp in the oocyte are a likely origin for Dpp signaling in the stretched and centripetally migrating follicle cells, respectively (Figure 3D’,H,I).

Although it is intriguing to observe such apparent dramatic changes in expression patterns, conservation of phenotype in face of substantial changes in the architecture of developmental programs has been widely reported and discussed under the concept of developmental systems drift [61]. In this particular case, it is important to remember that the functional event, the actual Dpp signal, remains a cooperative act between the ligand and its receptors. Thus, the selective pressure for Dpp function will be on this signaling event, as opposed to the source of the ligand. Interestingly, a similar interaction between germ line and soma has been described regarding Dpp signaling in the honeybee Apis mellifera [62]. In this system, dpp mRNA is localized to a dorsal stripe in the oocyte, and signaling activity is observed in the overlying follicle cells. While the absence of data on dpp expression in other closely related dipteran species precludes a clear evolutionary interpretation of these patterns, it does suggest that dpp expression in the follicle cells is a recent adaptation. A possible reason could be to prevent Dpp from remaining in the perivitelline cleft at the end of oogenesis, which could interfere with future embryonic dorsoventral patterning in which Dpp plays a large role.

### Regulation of *mirr*

One of the most interesting and salient aspects of this model system is the intimate genetic link between the novel phenotype—the dorsal appendages—and an ancestral and vital feature of embryonic development—dorsoventral polarity. One element of the network draws specific attention: the transcription factor *mirr*or (*mirr*). *mirr* regulates both the expression of *pipe* (*pip*), the gene encoding a sulfotransferase that is pivotal in providing dorsoventral polarity to the embryo, and broad (br), the gene that defines the dorsal appendage primordia. Our results show that *pip* expression is conserved, while its upstream regulator *mirr* appears to be part of the novel branch of the network in D. melanogaster. This observation suggests that *mirr*, rather than Br, operates as the key node of the network underlying the evolution of dorsal appendages.

Understanding the regulation of *mirr* in D. melanogaster then is necessary to understand how *mirr* could have been co-opted to regulate br, and possibly *pip*, in a novel manner. The best substantiated link between EGFr activation and *mirr* expression is the HMG-box transcription factor Capicua (Cic). Cic is a repressor of *mirr* in ventral and lateral follicle cells, and is downregulated in response to EGFr signaling [63,64]. However, global de-repression of *mirr* through Cic loss-of-function only results in visible expression of *mirr* in anterior follicle cells [65]. This observation suggests the additional involvement of Dpp activity in *mirr* regulation [31].

However, it has recently become clear that detectable *mirr* may not fully represent *mirr* activity throughout the follicular epithelium. Indeed, local de-repression of *mirr* through follicle cell clones in the posterior part of the epithelium is still sufficient to repress *pip*[40]. Interestingly, *pip* expression is unaffected when the Dpp pathway is disrupted [30]. Moreover, computational analyses have shown that the two-dimensional EGFr signaling profile is sufficient to explain the *pip* expression pattern, without any additional requirements for Dpp or other factors [66].

Thus, the fact that *mirr* expression is not seen with *in situ* hybridization does not preclude its activity in the follicular epithelium at a level sufficient to repress *pip*. In other words, we cannot conclude that *mirr* expression is absent in C. capitata from our data alone. However, although low (undetectable) *mirr* expression may be sufficient in D. melanogaster for regulation of *pip*, high (detectable) expression levels are necessary for activating the late enhancer of br and defining the dorsal appendage fate [31,32,65]. These high levels of *mirr* expression are clearly absent in C. capitata, and constitute a novel expression pattern related to the formation of a novel trait. Expression of br also depends on input from the Dpp pathway [22,30].

**Figure 5:**
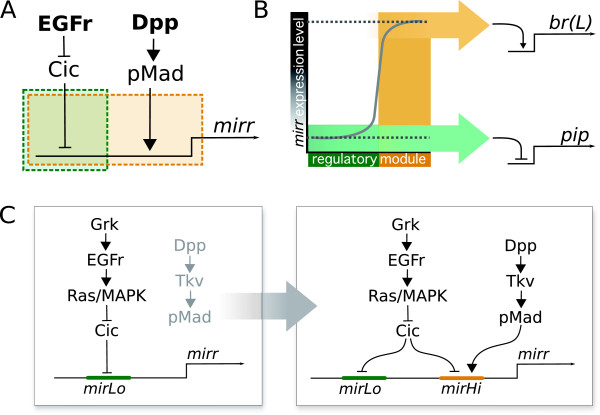
**A proposal for the evolution of *mirr*regulation in *D. melanogaster*.** (A) Two separate input modules regulate *mirr* expression: the green module uses only the input of the EGFr pathway via Cic, whereas the orange module requires both EGFr and Dpp input. (B) The two regulatory modules drive different expression levels of *mirr*. *pip* repression requires only low levels of *mirr*, which are provided through the green module, whereas the br(L) enhancer is activated only when *mirr* levels are sufficiently high, which is achieved through the orange module. (C) A proposal for the evolution of two regulatory modules, using two enhancers. A single enhancer (*mirLo*) is responsive to EGFr signaling only, and is sufficient to provide the low level of *mirr* expression required to repress *pip* as part of an ancestral signaling cassette. A second enhancer (*mirHi*) has evolved in Drosophila, which now drives *mirr* expression in response to both EGFr and Dpp signaling, in high levels that are sufficient for the activation of the br(L) enhancer.

Based on this data and our observations in C. capitata we propose a model that separates the contribution of *mirr* to dorsoventral polarity from its function in epithelial patterning, using two regulatory modules in *mirr* to obtain two distinct levels of expression (Figure 5A,B). One of these, responding only to EGFr activation—presumably through Cic down-regulation—is sufficient to generate the low expression levels required to repress *pip* (and likely also act on the early enhancer of br). Conversely, the other module requires Dpp signaling in addition to Cic down-regulation, and is able to regulate *mirr* expression to the high levels observed with *in situ* hybridization in the dorsal-anterior follicle cells of wild-type Drosophila egg chambers.

With this model we predict that dorsal de-repression of *mirr* through Cic is sufficient for *pip* repression, and constitutes an ancestral signaling cassette linking EGFr activation to embryonic dorsoventral patterning. Due to a non-cross-reactive antibody we were unable to confirm whether the localization of Cic in the C. capitata follicular epithelium fits our model, but we do note that cic mRNA is expressed in the egg chambers (Additional file 1).

Alternatively, *mirr* expression could be absent in C. capitata altogether, and *pip* could be regulated by another transcription factor. However, given the important role of *pip* in embryonic dorsoventral axis determination, and the dramatic defects that are caused with minimal variation in factors along the anteroposterior axis [67,68], we consider it likely that *pip* regulation happens through a conserved mechanism involving *mirr*. Pending the completion of the C. capitata whole genome sequencing project, it will be possible to test this hypothesis with a search for *mirr*-responsive elements in a Cc-*pip* regulatory region.

Presenting both modules as enhancers of *mirr* provides us with a hypothesis regarding their evolution (Figure 5C). The predicted ‘*mirLo*’ enhancer is expected to be ancestral, as we base its existence on the *mirr* and *pip* expression patterns in C. capitata egg chambers. *mirLo* would drive *mirr* expression in dorsal follicle cells in a level sufficiently high to repress *pip*, thus regulating dorsoventral polarity of the future embryo, downstream of EGFr signaling and independent of Dpp. The appearance of the second enhancer ‘*mirHi*’ would allow *mirr* to start responding to information from the Dpp pathway, and open up the evolutionary road to new patterns on the follicular epithelium.

## Conclusions

In the evolution of dorsal appendages, several genes have been co-opted into a network that originally regulated only dorsoventral polarity, using the input from a second signaling pathway active in the tissue. The activity of these pathways, EGFr and Dpp, defined a coordinate system on the epithelium upon which novel gene expression patterns were built. We have shown here how this coordinate system predates the evolution of dorsal appendages, providing positional information to the follicular epithelium that played a crucial role in future pattern formation.

The main regulators in this novel genetic network are transcription factors *mirr*, Pnt and Br. The latter integrates the information from upstream *mirr* and Pnt to specify the appendage primordia, and drives morphogenesis of the appendage [32,57]. Interestingly, while all three transcription factors have co-opted novel expression patterns and interactions to provide the main regulatory information for the epithelial positional cues to be translated into a novel morphology, both br, pnt and very likely *mirr* were already expressed in the ancestral non-appendage-forming epithelium. In this case it is notable that evolution may have taught ‘old genes new tricks’ [69] within the same broad spatial and developmental context. A novel morphological feature has been achieved by modifying the levels of existing expression patterns allocated to ancient developmental roles (br and likely *mirr*) with new enhancers responding to new information, or through the addition of an expression domain at another end of the same epithelium (pnt).

Most likely, the transcription factors Br, *mirr* and Pnt have not been the only ones to evolve new roles. Future research could go into the EGFr feedback loop, looking at rho and aos, as well as other EGFr targets. However, while the EGFr feedback loop was long thought to be the main patterning component of the eggshell [35], it has meanwhile been shown that eggshell patterning functions normally without several elements of this feedback system [70,71]. We can therefore conclude that changes in the regulation of *mirr*, pnt and br played important roles in the evolution of this novel morphology, and the detailed dissection of its molecular genetic basis constitutes the most important and immediate research agenda in the comprehension of the evolution of this morphological novelty.

Finally, we would like to stress that, as a derived organism, the model system D. melanogaster provides us with an excellent handle to tackle the question of evolutionary novelties, and for the dissection of the molecular mechanisms that underlie the process of co-option. We propose that many other traits that define Drosophila as derived—be they morphological or behavioural in nature—can be amenable to study using a comparative approach with other emergent dipteran model systems.

## Abbreviations

PCR: polymerase chain reaction; PBS: phosphate-buffered saline; ISH: *in situ* hybridization; FISH: fluorescent *in situ* hybridization.

## Competing interests

The authors declare that they have no competing interests.

## Authors’ contributions

ES conceived and designed the project, and wrote the paper; BMIV participated in the project design, performed the experiments, and wrote the paper; SR and JAL were invaluable for the execution of the experiments and the interpretation of results. All authors read and approved the final manuscript.

## Supplementary Material

Additional file 1 **Preliminary results: expression patterns of*Cc-rho*,*Cc-pnt*and*Cc-cic*.** Rhomboid: stage 9 and stage 10 ovaries do not show expression of the gene rhomboid in Ceratitis capitata. Positive control for the protocol and the probe is shown in a stage 9 to 10 embryo (ventral view).
Pointed: Stage 9 clearly shows the familiar posterior expression of pointed, which persists in stage 10 to 11 ovaries of Ceratitis capitata. No expression at the dorsal-anterior side of egg chambers could be detected. Fibers surrounding the egg chamber sometimes obscure the results, but changing the focal plane can confirm the identity of a signal as either coming from aspecific staining in fibers or staining of the cells in the follicular epithelium.
Capicua: Strong expression of capicua is seen in the nurse cells, but weaker expression can also be seen in the follicular epithelium (stage 10 egg chamber, below). Click here for file
