# Supplementary material for: Co-option of a coordinate system defined by the EGFr and Dpp pathways in the evolution of a morphological novelty
Source: EvoDevo. 2013 Mar 1;4:7. doi: 10.1186/2041-9139-4-7 (PMC3621409; doi:10.1186/2041-9139-4-7)

Data not shown in the figures beloning to Vreede *et al.*, "Co-option of a coordinate system defined by the EGFr and Dpp pathways in the evolution o a morphological novelty".

Preliminary results from *in situ* hybridization with riboprobes against *Cc-rhomboid*, *Cc-pointed* (common part of both isoforms), *Cc-capicua*.

Rhomboid: stage 9 and stage 10 ovaries do not show expression of the gene *rhomboid* in *Ceratitis capitata*. Positive control for the protocol and the probe is shown in a stage 9-10 embryo (ventral view).

Pointed: Stage 9 clearly shows the familiar posterior expression of *pointed*, which persists in stage 10-11 ovaries of *Ceratitis capitata*. No expression at the dorsal-anterior side of egg chambers could be detected. Fibres surrounding the egg chamber sometimes obscure the results, but changing focal plane can confirm the identity of a signal as either coming from aspecific staining in fibres, or staining of the cells in the follicular epithelium.

Capicua: Strong expression of *capicua* is seen in the nurse cells, but weaker expression can also be seen in the follicular epithelium (stage 10 egg chamber, below).

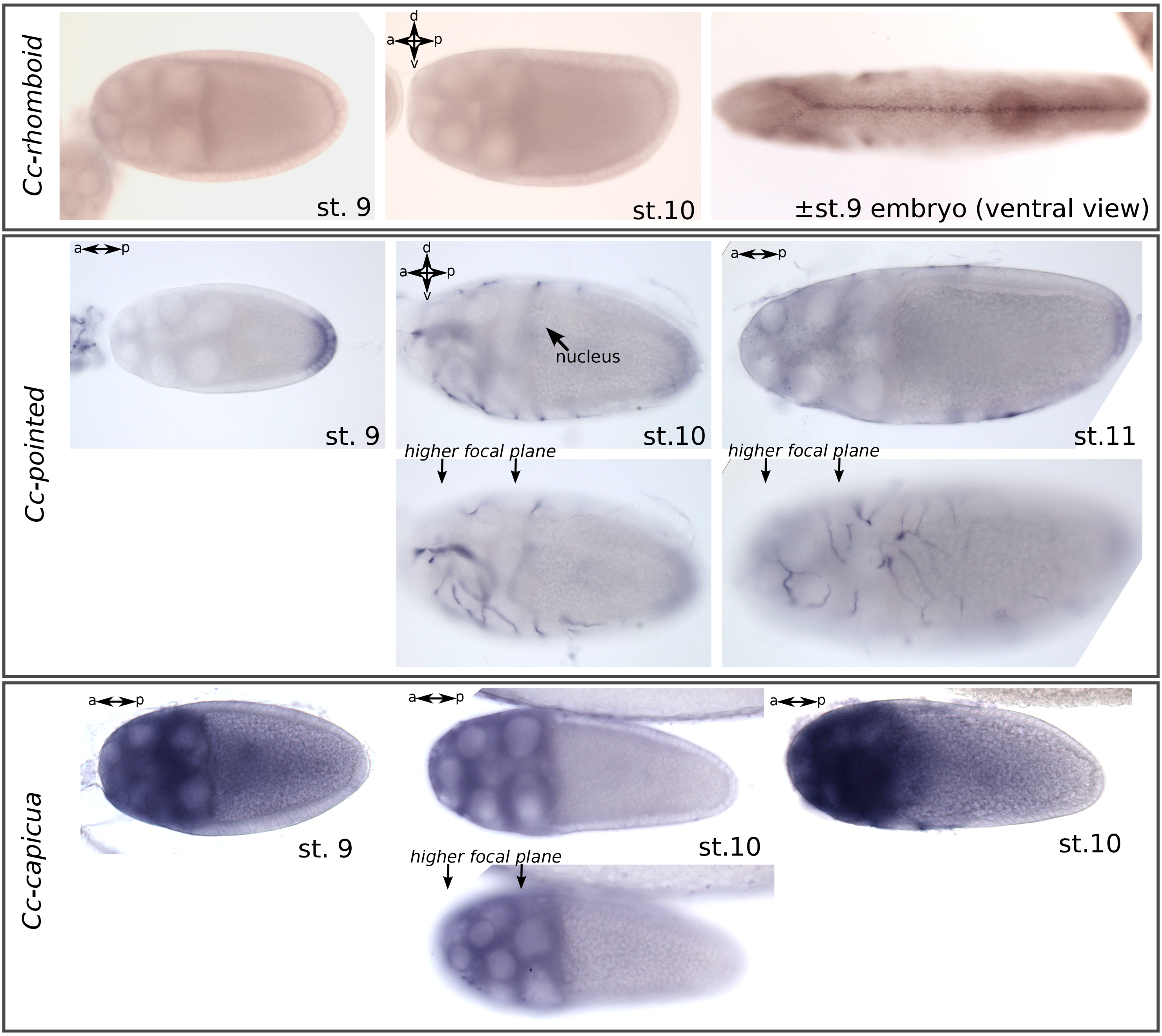

Supplement: Additional file 1 — Preliminary results: expression patterns ofCc-rho,Cc-pntandCc-cic. Rhomboid: stage 9 and stage 10 ovaries do not show expression of the gene rhomboid in Ceratitis capitata. Positive control for the protocol and the probe is shown in a stage 9 to 10 embryo (ventral view). Pointed: Stage 9 clearly shows the familiar posterior expression of pointed, which persists in stage 10 to 11 ovaries of Ceratitis capitata. No expression at the dorsal-anterior side of egg chambers could be detected. Fibers surrounding the egg chamber sometimes obscure the results, but changing the focal plane can confirm the identity of a signal as either coming from aspecific staining in fibers or staining of the cells in the follicular epithelium. Capicua: Strong expression of capicua is seen in the nurse cells, but weaker expression can also be seen in the follicular epithelium (stage 10 egg chamber, below). [file 2041-9139-4-7-S1.pdf]
